# Association Between Gestational Weight Gain and Obstetric Anal Sphincter Injury

**DOI:** 10.1097/og9.0000000000000117

**Published:** 2025-10-09

**Authors:** Sahar Khademioore, Sara Khademioureh, Ahmad Sofi-Mahmudi, Roxana Geoffrion, Rohan D'Souza, Giulia M. Muraca

**Affiliations:** Department of Health Research Methods, Evidence, and Impact, and the Department of Obstetrics and Gynecology, Faculty of Health Sciences, McMaster University, Hamilton, Ontario, the School of Public Health, University of Alberta, Edmonton, Alberta, and the Department of Obstetrics and Gynaecology, Faculty of Medicine, University of British Columbia, Vancouver, British Columbia, Canada; and the Clinical Epidemiology Unit, Department of Medicine, Solna, Karolinska Institutet, Stockholm, Sweden.

## Abstract

**OBJECTIVE:**

To investigate the association between gestational weight gain across all prepregnancy body mass index (BMI) categories and the risk of obstetric anal sphincter injury during vaginal birth.

**METHODS:**

Our retrospective population-based cohort study included individuals with singleton, term, vaginal births in the United States (2021–2023) and used data from the National Vital Statistics System's natality files. We examined the association between gestational weight gain (below, within, or above Institute of Medicine guidelines) and obstetric anal sphincter injury across BMI categories using logistic regression models. We assessed nonlinear associations between gestational weight gain and obstetric anal sphincter injury using restricted cubic spline regression and conducted a mediation analysis to examine the influence of fetal birth weight.

**RESULTS:**

Obstetric anal sphincter injury occurred in 1.2% of births, with rates of 1.3% among those who gained above the recommendations, 1.2% among those who gained within the recommendations, and 1.0% among those who gained below the recommendations. Gestational weight gain above and within guideline recommendations were associated with increased odds of obstetric anal sphincter injury among individuals with average-weight (adjusted odds ratio [aOR] 1.08; 95% CI, 1.05–1.10), overweight (aOR 1.13; 95% CI, 1.09–1.17), class I obesity (aOR 1.12; 95% CI, 1.06–1.18), and class II obesity (aOR 1.15; 95% CI, 1.06–1.26) BMIs. Gestational weight gain below and within recommendations showed a protective effect against obstetric anal sphincter injury only in individuals with average-weight BMIs (aOR 0.91; 95% CI, 0.88–0.93). Mediation analyses showed that gestational weight gain indirectly affected obstetric anal sphincter injury risk through birth weight across all BMI groups; direct effects were observed only in individuals with underweight, average-weight, and overweight BMIs.

**CONCLUSION:**

Gestational weight gain above the Institute of Medicine guideline was associated with increased risk of obstetric anal sphincter injury across all BMI categories except for underweight and class III obesity; weight gain below the guideline appeared to be protective only at average weight. These associations were largely mediated through birth weight.

## Abstract 2

Maintaining recommended gestational weight gain may reduce the risk of severe perineal tears during childbirth across most prepregnancy weight categories.

Obstetric anal sphincter injuries, or third- or fourth-degree perineal lacerations, are tears that extend through the external anal sphincter, internal anal sphincter, or rectal mucosa during vaginal birth.^1^ Obstetric anal sphincter injury is associated with physical complications such as infection, perineal pain, anal incontinence, sexual dysfunction, as well as negative effects on mental health.^2,3^ Although several other risk factors for obstetric anal sphincter injury have been identified, including primiparity, assisted vaginal birth, maternal race, and fetal macrosomia,^4–7^ the role of maternal weight–related factors remains unclear.

Gestational weight gain and prepregnancy body mass index (BMI, calculated as weight in kilograms divided by height in meters squared) are modifiable determinants of maternal health outcomes.^8^ The Institute of Medicine (IOM, now known as the National Academy of Medicine) provided recommended ranges of total weight gain based on prepregnancy BMI categories.^9^ The American College of Obstetricians & Gynecologists also endorses the IOM recommendations for weight gain during pregnancy.^10^ Although previous studies have examined the association between prepregnancy BMI and obstetric anal sphincter injury risk with inconsistent results, suggesting either a protective effect of higher prepregnancy BMI on risk of obstetric anal sphincter injury,^11–15^ or no statistically significant association,^7,16–18^ they did not consider gestational weight gain. Similarly, limited evidence suggests that excessive gestational weight gain based on IOM guidelines may increase obstetric anal sphincter injury risk,^19–21^ but the association between obstetric anal sphincter injury and weight gain within IOM recommendations is uncertain.^22^ Although the biological mechanism that links prepregnancy BMI and obstetric anal sphincter injury remains unclear, understanding the role of gestational weight gain may provide valuable insights into the pathways between maternal body composition, tissue biomechanics, and pelvic floor trauma during childbirth.

Our objective was to investigate the association between gestational weight gain and obstetric anal sphincter injury during vaginal birth across prepregnancy BMI categories. We assessed these associations by classifying gestational weight gain continuously and categorically based on the IOM recommendations. We hypothesized that gestational weight gain above IOM guidelines would increase the risk of obstetric anal sphincter injury and that gestational weight gain below guidelines would decrease the risk, with this association potentially differing across prepregnancy BMI groups and following a nonlinear pattern.

## METHODS

We conducted a retrospective population-based cohort study of live births from 2021 to 2023 in the United States. Data were obtained from the National Vital Statistics System natality files.^23^ The natality files data are derived from birth certificates registered in all 50 states and the District of Columbia, capturing more than 99% of births across the United States.^24^ Because we used publicly available de-identified data, ethics approval was not required. Power calculation details are available in Appendix 1, available online at http://links.lww.com/AOG/E350.

We included all singleton term pregnancies (37–42 weeks of gestation) that resulted in vaginal births in a hospital setting in the United States between January 1, 2021, and December 31, 2023. We excluded individuals who experienced weight loss during pregnancy, were younger than 15, or were older than 50 years. We also excluded observations with missing data on gestational weight gain, prepregnancy BMI, obstetric anal sphincter injury, or key covariates. Because the natality files recode weight loss during pregnancy as 0 in the “Weight Gain” variable, we could not distinguish between true 0 gain and weight loss.^25^ Therefore, individuals with 0 gain were excluded.

The exposure of interest was gestational weight gain, which is calculated by subtracting maternal weight at delivery from prepregnancy weight. Prepregnancy weight was self-reported by pregnant individuals in the Mother's Worksheet where individuals responded to the question, “What was your prepregnancy weight, that is, your weight immediately before you became pregnant with this child?”^24^ Acceptable ranges of prepregnancy weight were 50 to 400 pounds, and other values were coded as “not stated” in the data. Individuals' weight at delivery was collected in the Facility Worksheet. Accepted ranges of weight at delivery were 100 to 450 pounds, with other values recoded as “not stated.”^25^ The IOM recommendations for gestational weight gain are based on prepregnancy BMI category: underweight (BMI lower than 18.5: 28–40 lbs), average weight (BMI: 18.5–24.9: 25–35 lbs), overweight (BMI: 25.0–29.9: 15–25 lbs), and obesity (BMI 30 or higher: 11–20 lbs).^9^ The IOM guidelines do not have a distinct recommendation for each obesity class (class I obesity [BMI 30–34.9], class II obesity [BMI 35–39.9], and class III obesity [BMI 40 or higher]),^26^ and we considered the same recommended range for gestational weight gain for all obesity classes (11–20 lbs). Gestational weight gain also was categorized into three strata: below, within, and above the IOM recommendations for gestational weight gain. *Obstetric anal sphincter injury* was defined as third- or fourth-degree perineal laceration.

Potential confounding variables were identified a priori based on prior literature, consultation with clinicians and by creating a directed acyclic graph (Appendix 2, available online at http://links.lww.com/AOG/E350).^27^ These variables included: 1) maternal sociodemographic factors such as age (years), self-reported race and ethnicity (Hispanic, non-Hispanic Asian, non-Hispanic Black, non-Hispanic White, and additional races and ethnicities including non-Hispanic with more than one race, non-Hispanic Native Hawaiian or Other Pacific Islander, and non-Hispanic American Indian or Alaska Native), education level (high school or general educational development completed or less, some college credit, associate degree, bachelor’s degree, or graduate degree), health insurance status (Medicaid, private, self-pay, or other), and receipt of WIC (Special Supplemental Nutrition Program for Women, Infants, and Children) during pregnancy (yes or no) as a proxy for income level; 2) obstetric history factors such as parity (1, 2, 3 or more) and use of assisted reproductive technology (yes or no); 3) maternal complications such as preexisting and gestational hypertension, preexisting and gestational diabetes, and smoking before pregnancy (all yes or no); and 4) current pregnancy characteristics such as gestational age at delivery (completed weeks) and number of prenatal visits (continuous). Race and ethnicity were included in our model given evidence of racial and ethnic disparities in obstetric anal sphincter injury and gestational weight gain.^5,28^

Descriptive statistics were calculated for maternal characteristics and pregnancy outcomes, categorized based on gestational weight gain as below, within, or above the IOM recommendations. Logistic regression models were fitted for each prepregnancy BMI category, with obstetric anal sphincter injury as the outcome. The primary exposure was categorical gestational weight gain (below, within, and above IOM guideline recommendations), with gestational weight gain within the recommendations set as the reference category. The models were adjusted for potential confounders as described above. Odds ratios with 95% CIs were calculated and reported for unadjusted and adjusted models. Multicollinearity among covariates was assessed using generalized variance inflation factors. Generalized variance inflation factor values across all BMI categories ranged from 1.001 to 1.404, indicating no substantial multicollinearity concerns among the covariates.

To assess the potential nonlinear relationship between gestational weight gain as a continuous variable and obstetric anal sphincter injury, we employed logistic regression with restricted cubic spline terms for weight gain for each BMI category. These models allowed for flexible modeling of the exposure–outcome relationship while controlling for the same set of confounders previously listed. Four knots were placed at the 0.05, 0.35, 0.65, and 0.95 percentiles of the gestational weight gain distribution within each BMI category.^29^

We compared the characteristics of participants excluded due to missing values with those included in the final analysis and calculated standardized mean differences. To assess whether the association between gestational weight gain and obstetric anal sphincter injury varied by mode of delivery, we conducted stratified analyses by delivery type. Effect modification by delivery type was tested by including interaction terms between gestational weight gain categories and mode of delivery in both unadjusted and adjusted logistic regression models. When significant interactions were detected, we examined the association between gestational weight gain categories and obstetric anal sphincter injury for each mode of delivery (spontaneous vaginal, forceps, and vacuum delivery) using separate logistic regression models in each BMI category. All models were adjusted for the same set of covariates as the main model.

To examine whether and to what extent birth weight mediates the association between gestational weight gain and obstetric anal sphincter injury, we conducted a mediation analysis, using linear regression for the mediator (birth weight, continuous variable in grams) and logistic regression for obstetric anal sphincter injury. The analysis was performed separately for each BMI category, comparing gestational weight gain above and below recommendations with gestational weight gain within recommendations. We used a nonparametric bootstrap approach with 1,000 simulations to estimate the indirect effect of birth weight, the average direct effect (the association of gestational weight gain and obstetric anal sphincter injury while controlling for birth weight), and total effect (association between gestational weight gain and obstetric anal sphincter injury), adjusted for the same set of variables in our main models. The indirect effect, direct effect, and total effect were presented as risk differences (RDs) with 95% CIs derived from the bootstrap procedure. The proportion mediated was calculated to determine the relative contribution of the indirect pathway through birth weight using the Mediation package in R.^30^

All statistical analyses were performed using R 4.4.1.^31^ Statistical significance was assessed at *P*=.05.

## RESULTS

**Fig. 1.**
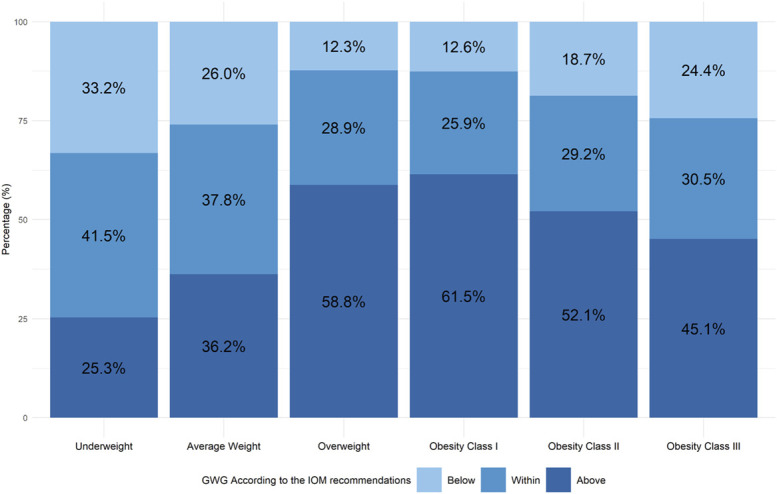
Proportion of gestational weight gain (GWG) below, within, and above the Institute of Medicine (IOM) guidelines in each prepregnancy body mass index (BMI) category among vaginal births, United States, 2021–2023 (N=5,942,164).

There were 10,951,038 live births from 2021 to 2023 in the United States. After applying the inclusion criteria and excluding individuals with missing values on the study covariates, the final cohort included 5,942,164 births (Appendix 3, available online at http://links.lww.com/AOG/E350). In this cohort, 2,822,701 (47.5%) gained weight above the recommendations, 1,944,479 (32.7%) gained within the recommendations, and 1,174,984 (19.8%) gained below the recommendations (Table 1; Appendix 4, available online at http://links.lww.com/AOG/E350). Individuals with prepregnancy BMIs in the overweight and obesity categories had higher proportions of weight gain above IOM guidelines (overweight: 58.8%; obesity class I: 61.5%; obesity class II: 52.1%; obesity class III: 45.1%) compared with individuals with average-weight BMIs (36.2%) and underweight BMIs (25.3%) (Fig. 1). Obstetric anal sphincter injury occurred in 1.2% (71,886) of the total population, with an incidence of 1.3% among those with weight gain above the guideline’s recommendations, compared with 1.2% among those within recommendations and 1.0% in those below recommendations (Table 1). Obstetric anal sphincter injury decreased with increasing BMI across almost all gestational weight gain categories (Appendix 5, available online at http://links.lww.com/AOG/E350).

**Table 1.** Sociodemographic and Pregnancy Characteristics of Individuals With Singleton, Term, Vaginal Births by Gestational Weight Gain Category According to Institute of Medicine Guidelines, United States, 2021–2023

Characteristic	GWG Category According to the IOM Guideline			Total (N=5,942,164)	SMD
	Below (n=1,174,984)	Within (n=1,944,479)	Above (n=2,822,701)		
Age (y)	28.9±5.9	29.3±5.6	28.7±5.6	28.9±5.7	0.071
15–19	65,921 (5.6)	81,354 (4.2)	134,603 (4.8)	281,878 (4.7)	0.086
20–29	556,605 (47.4)	889,685 (45.8)	1,415,098 (50.1)	2,861,388 (48.2)	
30–39	514,393 (43.8)	913,750 (47.0)	1,202,352 (42.6)	2,630,495 (44.3)	
40–49	38,065 (3.2)	59,690 (3.1)	70,648 (2.5)	168,403 (2.8)	
Maternal race and ethnicity					
Hispanic	347,682 (29.6)	516,075 (26.5)	680,763 (24.1)	1,544,520 (26.0)	0.194
Non-Hispanic Asian	103,505 (8.8)	143,944 (7.4)	114,185 (4.0)	361,634 (6.1)	
Non-Hispanic Black	161,977 (13.8)	220,840 (11.4)	354,334 (12.6)	737,151 (12.4)	
Non-Hispanic White	524,710 (44.7)	1,005,576 (51.7)	1,567,368 (55.5)	3,097,654 (52.1)	
Additional races and ethnicities*	37,110 (3.2)	58,044 (3.0)	106,051 (3.8)	201,205 (3.4)	
Maternal education					
High school or high school equivalent completed or less	510,087 (43.4)	694,186 (35.7)	1,027,494 (36.4)	2,231,767 (37.6)	0.129
Some college credit, Associate’s degree, or Bachelor's degree	515,175 (43.8)	942,384 (48.5)	1,423,364 (50.4)	2,880,923 (48.5)	
Graduate	149,722 (12.7)	307,909 (15.8)	371,843 (13.2)	829,474 (14.0)	
Marital status					
Married	595,923 (57.9)	1,086,297 (63.6)	1,491,997 (58.8)	3,174,217 (60.2)	0.077
Not married	433,136 (42.1)	622,882 (36.4)	1,045,922 (41.2)	2,101,940 (39.8)	
WIC support received during pregnancy	398,342 (33.9)	560,270 (28.8)	849,792 (30.1)	1,808,404 (30.4)	0.073
Smoker before pregnancy	51,832 (4.4)	67,928 (3.5)	133,731 (4.7)	253,491 (4.3)	0.042
Infertility treatment	18,265 (1.6)	35,155 (1.8)	43,784 (1.6)	97,204 (1.6)	0.013
Prepregnancy diabetes	8,222 (0.7)	11,236 (0.6)	16,765 (0.6)	36,223 (0.6)	0.010
Prepregnancy hypertension	21,064 (1.8)	33,847 (1.7)	63,254 (2.2)	118,165 (2.0)	0.024
Parity					
1	416,237 (35.4)	744,657 (38.3)	1,250,397 (44.3)	2,411,291 (40.6)	0.127
2	382,887 (32.6)	635,466 (32.7)	839,602 (29.7)	1,857,955 (31.3)	
3 or more	375,860 (32.0)	564,356 (29.0)	732,702 (26.0)	1,672,918 (28.2)	
Gestational age at delivery (wk)	38.8±1.0	38.9±1.0	39.0±1.1	38.9±1.1	0.124
37–38	430,927 (45.2)	609,519 (40.2)	822,134 (38.1)	1,862,580 (40.3)	0.108
39–41	520,297 (54.6)	903,742 (59.6)	1,327,187 (61.6)	2,751,226 (59.5)	
42 or more	1,969 (0.2)	3,784 (0.2)	6,389 (0.3)	12,142 (0.3)	
Primary payer					
Medicaid	540,231 (46.0)	754,097 (38.8)	1,133,880 (40.2)	2,428,208 (40.9)	0.113
Private insurance	547,610 (46.6)	1,061,745 (54.6)	1,517,776 (53.8)	3,127,131 (52.6)	
None of the above^†^	87,143 (7.4)	128,637 (6.6)	171,045 (6.1)	386,825 (6.5)	
No. of prenatal visits	10.6±4.2	11.2±3.9	11.5±3.8	11.2±3.9	0.139
Gestational diabetes	105,755 (9.0)	136,621 (7.0)	167,262 (5.9)	409,638 (6.9)	0.078
Gestational hypertension	632,222 (5.4)	123,987 (6.4)	284,855 (10.1)	472,064 (7.9)	0.118
Induction of labor					
Yes	425,616 (36.2)	744,616 (38.3)	1,253,369 (44.4)	2,423,601 (40.8)	0.112
No	749,239 (63.8)	1,199,699 (61.7)	1,569,122 (55.6)	3,518,060 (59.2)	
Unknown	129 (0.0)	164 (0.0)	210 (0.0)	503 (0.0)	
Birth weight (g), mean	3,193.1±420.9	3,306.4±417.9	3,422.7±435.2	3,340.3±436.0	0.360
Less than 1,500	192 (0.0)	211 (0.0)	289 (0.0)	692 (0.0)	0.210
1,500–2,499	53,834 (4.6)	49,009 (2.5)	45,027 (1.6)	147,870 (2.5)	
2,500–3,999	1,085,524 (92.4)	1,798,123 (92.5)	2,517,598 (89.2)	5,401,245 (90.9)	
4,000 or more	35,434 (3.0)	97,136 (5.0)	259,787 (9.2)	392,357 (6.6)	
Method of delivery					
Spontaneous	1,125,029 (95.7)	1,858,868 (95.6)	2,693,314 (95.4)	5,677,211 (95.5)	0.016
Forceps	6,916 (0.6)	13,123 (0.7)	21,984 (0.8)	42,023 (0.7)	
Vacuum	43,039 (3.7)	72,488 (3.7)	107,403 (3.8)	222,930 (3.8)	
OASI	11,784 (1.0)	23,223 (1.2)	36,879 (1.3)	71,886 (1.2)	0.019

GWG, gestational weight gain; IOM, Institute of Medicine; SMD, standardized mean difference; WIC, (Special Supplemental Nutrition Program for Women, Infants, and Children); OASI, obstetric anal sphincter injury .

Data are mean±SD or n (column %) unless otherwise specified.

* Includes non-Hispanic individuals of multiple races, Native Hawaiian or Other Pacific Islander, and American Indian or Alaska Native; aggregated due to small numbers.

† Self-pay or other types of payment.

*Khademioore. Gestational weight gain and obstetric anal sphincter injury. O&G Open 2025*.

**Fig. 2.**
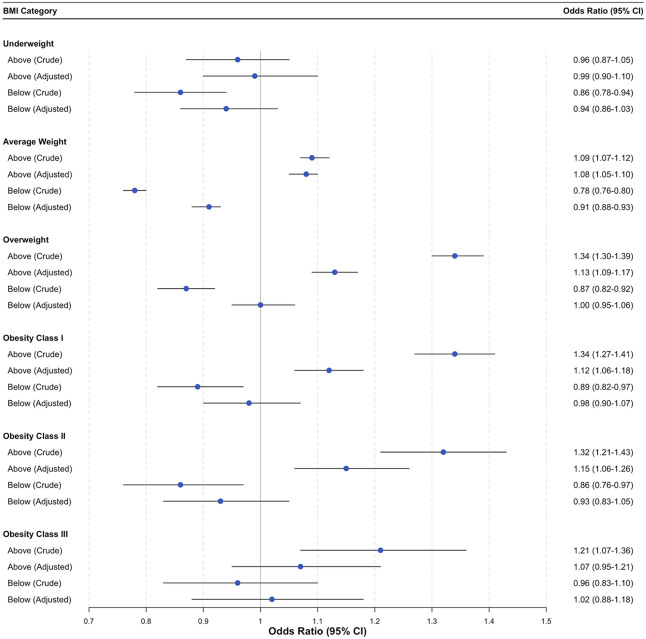
Association between gestational weight gain and obstetric anal sphincter injuries by prepregnancy body mass index (BMI) category. Forest plots show crude and adjusted odds ratios with 95% CIs. Adjusted models included maternal age, maternal race and ethnicity, maternal education level, payment source for delivery, receipt of WIC (Special Supplemental Nutrition Program for Women, Infants, and Children) during pregnancy, pre-existing hypertension, pre-existing diabetes, gestational diabetes, gestational hypertension, prepregnancy smoking, parity, use of assisted reproductive technology, gestational age at delivery, number of prenatal visits. Reference category: weight gain within guidelines.

Compared with weight gain within IOM recommendations, gestational weight gain above the guidelines was associated with increased odds of obstetric anal sphincter injury across most prepregnancy BMI categories. Specifically, individuals with average-weight BMIs had 8% increased odds (adjusted odds ratio [aOR], 1.08; 95% CI, 1.05–1.10), those with overweight BMIs had 13% increased odds (aOR 1.13; 95% CI, 1.09–1.17), those with class I obesity BMIs had 12% increased odds (aOR 1.12; 95% CI, 1.06–1.18), and those with class II obesity BMIs had 15% (aOR 1.15; 95% CI, 1.06–1.26) increased odds. However, no associations were observed for individuals with underweight or class III obesity BMIs (Fig. 2).

*Khademioore. Gestational weight gain and obstetric anal sphincter injury. O&G Open 2025*.

When comparing gestational weight gain below the recommendations with gestational weight gain within the recommendations, a reduction in the odds of obstetric anal sphincter injury was observed only among individuals with average-weight BMIs (aOR 0.91; 95% CI, 0.88–0.93). In all other BMI categories, no statistically significant associations were found after adjustment for confounders (Fig. 2; see Appendix 6, available online at http://links.lww.com/AOG/E350).

**Fig. 3.**
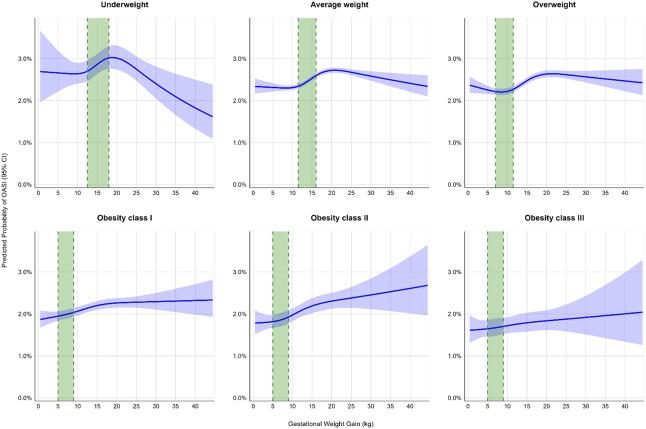
Association between gestational weight gain and obstetric anal sphincter injury (OASI) based on the restricted cubic spline regression models. Adjusted models included maternal age, maternal race and ethnicity, maternal education level, payment source for delivery, receipt of WIC (Special Supplemental Nutrition Program for Women, Infants, and Children) during pregnancy, pre-existing hypertension, pre-existing diabetes, gestational diabetes, gestational hypertension, prepregnancy smoking, parity, use of assisted reproductive technology, gestational age at delivery, number of prenatal visits. Data were fitted by a logistic regression model, and the models were conducted with four knots at the 0.05, 0.35, 0.65, and 0.95 percentiles of gestational weight gain. *Blue line* indicates predicted probability of OASI at each gestational weight gain value. *Blue shading* indicates 95% CIs around predictions. *Green shaded area* indicates Institute of Medicine–recommended gestational weight gain range for that body mass index (BMI) category.

When gestational weight gain was modeled as a continuous variable (Appendix 7 [unadjusted], available online at http://links.lww.com/AOG/E350, and Fig. 3 [adjusted]), there was an association between gestational weight gain and obstetric anal sphincter injury (*P*=.008; Fig. 3) among individuals with underweight BMIs. This association followed an inverted U-shaped pattern (*P*-value for nonlinearity=.003), with predicted probability increasing up to approximately 15–20 kg of weight gain before declining, although CIs widened at the extremes.

*Khademioore. Gestational weight gain and obstetric anal sphincter injury. O&G Open 2025*.

Among individuals with average-weight and overweight BMIs, there were associations between gestational weight gain and obstetric anal sphincter injury (*P*<.001 for both) with U-shaped pattern relationships (*P*-value for nonlinearity <.001 for both). In those with average-weight BMIs, there was an initial decrease in obstetric anal sphincter injury until 7–8 kg, followed by a steady increase in risk with higher weight gain. In individuals with overweight BMIs, the lowest predicted probability of obstetric anal sphincter injury was between 8 and 10 kg of weight gain, with increased risk at both lower and higher weight gains. For individuals with classes I and II obesity, there was an association between gestational weight gain and obstetric anal sphincter injury (both *P*<.001), which appeared linear in both groups (*P* for nonlinearity=.1 and .4, respectively), showing a gradual increase in obstetric anal sphincter injury risk with increasing gestational weight gain. There was no evidence of an association between gestational weight gain and obstetric anal sphincter injury risk in the obesity class III BMI group (*P*=.3) and no evidence of nonlinearity (*P*=.9).

When comparing individuals excluded from the final cohort due to missingness (n=500,124) and those included in final cohort (N=5,942,164), significant differences (standardized mean difference>0.1) were observed in the distribution of maternal race and ethnicity, number of prenatal visits, mean gestational age at delivery, smoking status, and WIC recipient status. All other clinical and obstetric characteristics showed minimal differences between the groups (Appendix 8, available online at http://links.lww.com/AOG/E350).

In the mode of delivery–stratified models, the association between gestational weight gain and obstetric anal sphincter injury varied between mode of delivery only in the average-weight BMI group (*P* for interaction=.03), where above-guideline gestational weight gain was associated with a higher rate of obstetric anal sphincter injury in the spontaneous vaginal delivery group (aOR 1.09; 95% CI, 1.06–1.12); no such association was observed in the forceps and vacuum groups (Table 2). Appendix 9, available online at http://links.lww.com/AOG/E350, represents the results of stratified analysis in crude models where the interactions were significant in the average-weight, overweight, and class I obesity BMI categories.

**Table 2.** Association Between Gestational Weight Gain and Obstetric Anal Sphincter Injury in Each Prepregnancy Body Mass Index Category by Mode of Delivery

GWG Category According to the IOM Guideline	Spontaneous Vaginal Delivery		Forceps Delivery		Vacuum Delivery	
	n/N (%)	Adjusted OR (95% CI)*	n/N (%)	Adjusted OR (95% CI)*	n/N (%)	Adjusted OR (95% CI)*
Average-weight BMI						
Below	5,571/633,352 (0.88)	0.90 (0.87–0.93)	542/4,267 (12.70)	0.87 (0.78–0.98)	1,574/26,797 (5.87)	0.94 (0.88–1.01)
Within	10,348/915,386 (1.13)	Ref	1,168/7,482 (15.61)	Ref	2,762/40,500 (6.82)	Ref
Above	10,953/873,932 (1.25)	1.09 (1.06–1.12)	1,325/8,386 (15.80)	1.00 (0.91–1.09)	2,648/40,636 (6.52)	1.00 (0.94–1.06)

OR, odds ratio; GWG, gestational weight gain; IOM, Institute of Medicine; BMI, body mass index; Ref, reference.

* Adjusted models included maternal age, maternal race and ethnicity, maternal education level, payment source for delivery, receipt of WIC (Special Supplemental Nutrition Program for Women, Infants, and Children) during pregnancy, preexisting hypertension, preexisting diabetes, gestational diabetes, gestational hypertension, prepregnancy smoking, parity, use of assisted reproductive technology, gestational age at delivery, and number of prenatal visits.

In individuals with underweight BMIs, gestational weight gain above the IOM recommendations was directly associated with a decreased risk of obstetric anal sphincter injury (risk difference [RD] 0.2%; Appendices 10–16, available online at http://links.lww.com/AOG/E350) compared with gestational weight gain within the recommendations; however, the total effect was nullified by the positive indirect effect of gestational weight gain through fetal birth weight. Among individuals with average-weight prepregnancy BMIs, the association between gestational weight gain and obstetric anal sphincter injury was fully explained by the mediating effects of birth weight. Although the direct effect on gestational weight gain above the guideline recommendations on obstetric anal sphincter injury was protective (RD −0.1%) compared with weight gain within the recommendations, the indirect effect through birth weight increased the risk of obstetric anal sphincter injury and was of greater magnitude (RD 0.2%). In the group with overweight BMIs, gestational weight gain below the recommendations directly increased the risk of obstetric anal sphincter injury (0.1%) compared with gestational weight gain within the recommendations, but this effect also was nullified due to the protective indirect effect of gestational weight gain through birth weight (−0.1%). There were no direct effects between gestational weight gain and obstetric anal sphincter injury in the groups with obesity classes I and II BMIs. The total effects were completely driven by the indirect effect through birth weight for the class I obesity BMI group; while 70% of the total effect was through birth weight for the class II obesity BMI group.

## DISCUSSION

We conducted a population-based cohort study of individuals with vaginal births in the United States to investigate the association between gestational weight gain and obstetric anal sphincter injury across different prepregnancy BMI categories. Our findings showed a consistent pattern of increased obstetric anal sphincter injury risk with excessive gestational weight gain compared with gestational weight gain within guideline recommendations, although the magnitude of this risk varied by prepregnancy BMI category. Conversely, weight gain below the guidelines showed a protective association in only the average-weight BMI category.

Mediation analysis allows for the decomposition of the total effect of an exposure (gestational weight gain) on an outcome (obstetric anal sphincter injury) into direct and indirect pathways, providing insight into potential causal mechanisms. The direct effect represents the association between gestational weight gain and obstetric anal sphincter injury that operates through pathways other than birth weight; the indirect effect quantifies the portion of the association that is mediated through birth weight. This approach enabled us to better understand the indirect effect of gestational weight gain mediated through birth weight, which, in comparison with the group of individuals whose weight gain fell within the guideline, consistently lowered the risk of obstetric anal sphincter injury for those in the group with weight gain below the guideline and increased the risk for those in the group with weight gain above the guideline. A direct effect of gestational weight gain on obstetric anal sphincter injury was restricted to the underweight, average-weight, and overweight BMI groups.

Our findings are consistent with those of a previous U.S. study that examined the relationship between gestational weight gain and obstetric anal sphincter injury within obesity classes using Bayesian modeling.^19^ The study reported a higher risk of perineal laceration with above-guideline gestational weight gain in individuals with obesity class I (aOR 1.16; 95% CI, 1.09–1.23) and class II (aOR 1.13; 95% CI, 1.03–1.25) BMIs, compared with gestational weight gain within the guidelines, while finding no significant associations for below-guideline gestational weight gain. Similarly, their results showed no significant associations in class III obesity, aligning with our adjusted models. Similarly, Ornaghi et al^21^ reported that excessive gestational weight gain, compared with adequate gestational weight gain, was associated with increased risk of obstetric anal sphincter injury (aOR 2.04; 95% CI, 1.12–3.76). Our study builds on these findings by examining the variability in these associations across BMI categories. In contrast, Kominiarek et al^22^ found no association between gestational weight gain above and within guideline recommendations and obstetric anal sphincter injury (aOR 1.04; 95% CI, 0.88–1.22). Their study did not stratify by BMI or adjust for prepregnancy BMI, which may have masked variations in gestational weight gain-related risk among BMI subgroups.

The observed nonlinear relationship between gestational weight gain and obstetric anal sphincter injury in underweight, average-weight, and overweight BMI categories represents a novel finding. This pattern suggests an optimal window for weight gain that minimizes obstetric anal sphincter injury risk, which may differ from current IOM guidelines. However, all maternal and child health outcomes should be considered when assessing an optimal range for gestational weight gain for each BMI category.

The RD of the total effect of gestational weight gain on obstetric anal sphincter injury ranged from −0.2% to 0.1%, depending on prepregnancy BMI and gestational weight gain category. The small absolute differences in this study population reflect the relatively low rate of obstetric anal sphincter injury, overall (1.1%). However, in populations with higher baseline rates of obstetric anal sphincter injury, such as among primipara, Asian patients, and those with forceps and vacuum delivery,^5,6^ the changes in risk may be more significant from clinical and public health perspectives. For example, in countries with greater use of forceps delivery, such as Canada, recent estimates of obstetric anal sphincter injury after forceps delivery approach 22%.^32^ In this population, a relative 18% decrease in risk (equivalent to the -0.2% RD in our study) translates to an absolute 4.0% reduction in obstetric anal sphincter injury (from 22% to 18.0%).

This study has several notable strengths. First, we analyzed gestational weight gain both categorically and continuously, which allowed us to detect nonlinear relationships. Further, by assessing gestational weight gain continuously and independent of the IOM guidelines categories, we recognized that these guidelines may not accurately capture the optimal gestational weight gain ranges for obstetric anal sphincter injury outcomes. Our findings also have implications for understanding how gestational weight gain influences obstetric anal sphincter injury risk through different pathway across BMI categories.

The limitations of our study include the lack of data on the rate of gestational weight gain throughout pregnancy. Rapid weight gain may affect tissue remodeling and increase obstetric anal sphincter injury risk.^33^ Our findings may have limited generalizability given the differences between included and excluded participants, particularly in terms of racial and ethnic composition, prenatal care utilization, gestational age at delivery, smoking, and WIC recipient status. Further, this study focused on gestational weight gain and excluded those with 0 gain or weight loss. The lack of information on episiotomy use in our data source further limits our comparison of obstetric anal sphincter injury risk. Data constraints precluded the analysis of the relationship between gestational weight gain and subcategories of obstetric anal sphincter injury based on the degree of laceration (third or fourth degree).

In this study, gestational weight gain above the IOM guidelines was associated with an increased risk of obstetric anal sphincter injury in the average-weight, overweight, and obesity classes I and II prepregnancy BMI categories; gestational weight gain below the recommendation showed a protective association only for individuals with average-weight BMIs. These associations appeared to be largely mediated through birth weight.
